# Diversity of human astroviruses in Germany 2018 and 2019

**DOI:** 10.1186/s12985-022-01955-3

**Published:** 2022-12-21

**Authors:** Sandra Niendorf, Andreas Mas Marques, Claus-Thomas Bock, Sonja Jacobsen

**Affiliations:** 1 Unit Viral Gastroenteritis and Hepatitis Pathogens and Enteroviruses, Department of Infectious Diseases, Robert Koch Institute, Seestraße 10, 13353 Berlin, Germany; 2 Consultant Laboratory for Noroviruses, Robert Koch Institute, Seestraße 10, 13353 Berlin, Germany

**Keywords:** Human astroviruses, Viral gastroenteritis, Molecular surveillance, Genotyping

## Abstract

Aim of this study was to investigate the molecular diversity of human astroviruses (HAstV) in Germany. A follow-up study was performed with human stool samples collected in 2018–2019, which were genotyped retrospectively. A total of 2645 stool samples, collected between January 2018 and December 2019 from sporadic cases and outbreaks of acute gastroenteritis were analyzed. An algorithm of PCR systems was used to characterize human astrovirus. Human astroviruses were found in 40 samples (positive rate: 1.6%). During the study period, children aged 1–2 years (48%) were most affected by HAstV. Genotyping revealed a number of nine circulating genotypes representing four human Mamastrovirus species. Strain MLB1 was predominant in the study population with a detection rate of 25% followed by HAstV1 with a positive rate of 20%. The diversity of astrovirus genotypes seems to be rather stable in Germany in the last years. A clustering of regionally and/or temporally linked human astroviruses in Germany was not detectable.

**Supplementary Information:**

The online version contains supplementary material available at 10.1186/s12985-022-01955-3.

## Introduction

Astroviruses are non-enveloped small round particles (~ 30 nm) with single-stranded positive sense RNA (ssRNA). The genome is ~ 7 kb in length and consists of a 5′ untranslated region with an attached viral genome-linked VPg protein and a poly(A) tail. The genome consists of three open reading frames (ORF) [1–3]. Open reading frame 1 (ORF1a and ORF1b) encodes non-structural proteins involved in RNA transcription and replication, and ORF2 encodes the viral capsid protein. A further ORF (ORFX) with an alternative start codon overlapping the 5′ end of ORF2 has been observed in classic HAstV viruses [4] and encodes the XP-protein. It is translated during virus infection and promotes an efficient virus assembly/release as shown in cell culture experiments [5].

Human astroviruses (HAstV) belong to the family of *Astroviridae*. According to the International Committee for the Taxonomy of Viruses (ICTV) proposal the genus Mamastrovirus (MAstV) is classified into 19 species [6]. To date, four MAstV species (MAstV-1, MAstV-6, MAstV-8 and MAstV-9) have been identified in humans. MAstV-1 consists of classic genotypes 1–8, among those HAstV-1 is the most dominate strain worldwide [2, 7–9]. The clade of classic astroviruses is genetically very different from MLB and VA clades, which are more closely related to animal astroviruses [10–12]. The MLB clade with strains MLB1, MLB2, and MLB3 was assigned to the MAstV-6 species. The VA clade consists of VA/HMO strains: VA-2, VA-4 and VA-5 which were grouped in MAstV-8 species, VA-1 and VA-3 belong to MAstV-9 species.

Transmission of HAstV occurs by fecal–oral route, mainly by person-to-person contact, food-, or water-borne transmission [2]. HAstV infections have been reported worldwide, especially in infants, children, elderly people and immunocompromised patients. Often, symptoms of mild watery diarrhea are observed. Less common signs and symptoms are vomiting, headache, fever, abdominal pain and anorexia. The infection is self-limiting within 2–4 days, but persistent infections in immunocompromised patients as well as asymptomatic infections in children have been described [13]. It is currently discussed whether human astrovirus has neurotropic potential especially in immunosuppressed/immunodeficient patients [14–16].

Little is known about the change of astrovirus diversity over time, most studies focus on infection of infant inpatients in short time intervals. Therefore, we conducted a follow-up study on the circulation of HAstV genotypes in Germany 2018–2019.

## Results and discussion

A total of 2645 stool samples were analyzed for the presence of HAstV in 2018 and 2019 in Germany (1165 and 1480 samples respectively). 43.5% of analyzed samples were from male patients and 51.7% were from females (gender information was not available for 126 samples = 4.8%, Table 1). RNA of human astroviruses were found in 40 (1.5%) stool samples in the study cohort, 16 positive samples were detected in 2018 (16/1165 = 1.4%) and 24 samples with HAstV were found in 2019 (24/1480 = 1.6%). The median age of the infected patients was 27 months (minimum: 6 months, maximum: 1082 months). While a higher positivity detection rate was found in females, this difference was not statistically significant (*p* = 0.0662, α = 0.05, Fisher’s exact test). Infants between 1 and 2 years of age were most affected by HAstV: in this age group 20 samples with HAstV infection were detected (total number of positive samples with age specification: 36 = detection rate of 56% (Table 1). Two samples were positive in the age group younger than 1 year and there was a significant difference between ages group < 1 and age group 1–2 years (*p* = 0.0006, α = 0.05, Fisher’s exact test), the latter having a higher positivity rate. Five positives samples were found in the age group of 3–4 years, and three positive samples in the age group between 5 and 9 years respectively. The positive rate in adults was lower than in children. In total six positive samples were found in adults (age groups of 30–39 years, 60–69 years and > 80 years).

**Table 1:** Characteristics of HAstV infections among patients

Epidemiological data	n = samples analyzed	No. of HAstV positive samples (%)
*Gender*		
Female	1368	23 (57.5)
Male	1151	14 (35)
No gender information	126	3 (7.5)
HAstV affected age group (years)*		
< 1	182	2 (1.1)
1–2	291	20 (6.9)
3–4	131	5 (3.8)
5–9	160	3 (1.9)
30–39	122	1 (0.8)
60–69	159	1 (0.6)
> 80	559	4 (0.7)

*4 samples without age statement were not included in this table = 36 positive samples with age statement

The detection rate of 1.4% was lower than in the long-term study in Germany, which was conducted from 2010 to 2015 (2–12.1% per year) and slightly lower than in Canada (2.1%) was found in pediatric patients (2014–2018), similar to the detection rate of 2.6% in pediatric patients in Thailand (2017–2020) [7, 17, 18].

From all 40 HAstV positive samples genotyping analysis was done. Nine different genotypes/strains were found in the study period: classic genotypes HAstV1, HAstV3-5, HAstV8 (MAstV-1 species), as well as variants MLB1, MLB2 (MAstV-6 species), and VA1, VA2 (MastV-9) (Table 2). Four sequences could only be genotyped as HAstV classic, because amplification of just a short fragment was possible. Variant MLB1 was the most frequent strain (10/40 samples = 25%) followed by HAstV1 (8/40 samples = 20%) and HAstV3 (5/40 samples = 12.5%). HAstV5, HAstV8 and VA2 were the least frequent genotypes in this study population with one positive sample each (2.5%). No recombination events were seen in HAstV classic viruses or in HAstV MLB variants circulating in Germany in 2018 or 2019. HAstV1, HAstV3 and unclassified classic genotypes as well as MLB1 and MLB2 were present in samples from 2018 and 2019, while HAstV5 and VA1 and VA2 could only be detected in 2019 (Table 2). MLB1 and MLB2 strains were identified in children, while classic genotypes and HAstV VA1 were also detected in adult patients (data not shown). Similar to the 2010–2015 study, the variability of HAstV genotypes was greatest in the age group: 1–2 years, with seven of nine genotypes detected [7]. In the study period 2018–2019, MLB1 viruses were detected in January, March, May, September, November and December while HAstV1 was found in samples collected in January, March, May, and November respectively. In January four different genotypes were detected: HAstV1, MLB1, HAstV classic and VA1, in February, March, May and November three different genotypes were detectable. In samples from the other months one or two different variants were detected in the patients.

**Table 2:** HAstV genotypes detected in 2018–2019

Collection period	HAstV 1	HAstV 3	HAstV 4	HAstV 5	HAstV 8	HAstV classic*	MLB1	MLB2	VA1	VA2	total
2018	4	2	2	0	0	3	5	1	0	0	17
2019	3	3	0	1	0	1	3	1	1	1	14
No collection date available	1	0	1	0	1	0	2	1	3	0	9
Total	8 (20%)	5 (12.5%)	3 (7.5%)	1 (2.5%)	1 (2.5%)	4 (10%)	10 (25%)	3 (7.5%)	4 (10%)	1 (2.5%)	40

*A sample was defined as HAstV classic if only a fragment of PCR screening assay could be sequenced (~ 170 bp), the short sequence cannot discriminate HAstV classic genotypes. From nine positive HAstV samples the collection date was not available

Nowadays astrovirus MLB strains are widely distributed in the human population. MLB1 was already detected in Germany 2013 but in the study period 2010–2015 HAstV1 was the most predominant virus [7]. VA strains were detected in German patients in samples from 2011 (VA1) and 2015 (VA2) whereas MLB2 was detectable from 2013 until 2015. These variants were still present in patients with gastroenteritis from 2018 to 2019. Novel HAstV strains (VA and MLB) were not exclusively correlated with gastroenteritis, they were also associated with asymptomatic infection among children in Spain indicating that there might be a protective mechanism of the presence of HAstV toward interference with other intestinal pathogens [13]. The novel HAstV genotypes were found in infants as well as adults in Germany 2018–2019.

**Fig. 1:**
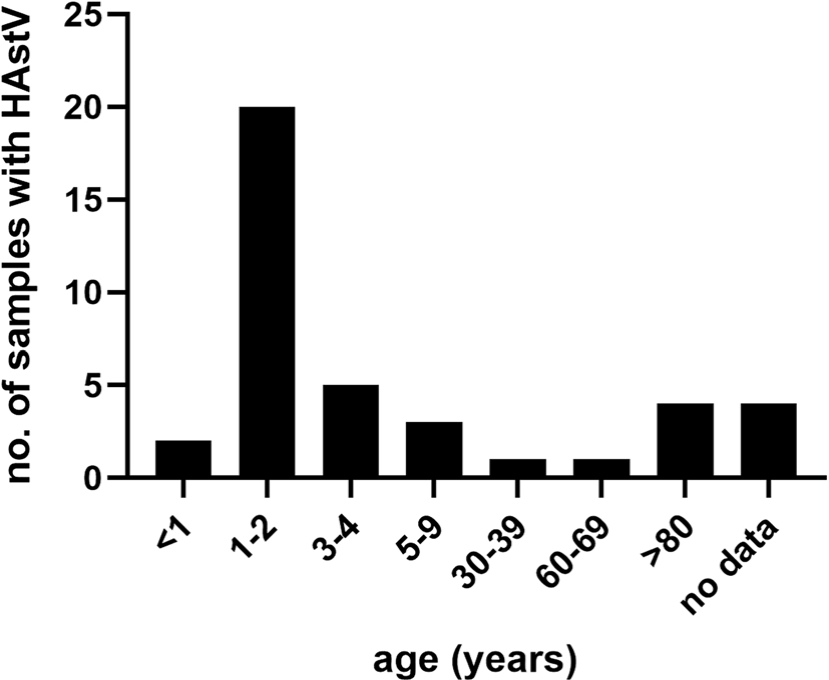
Number of HAstV positive samples in relation to collection date (month). Collection date was not available in 9 of 40 samples

In 2018–2019 HAstV was detected in every month except June and October (Fig. 1). Interestingly, a slightly higher number of positive samples in May were also seen in our data from 2010 to 2015 [7]. Due to temperate climate in Germany, patients were more affected by HAstV in winter.

**Fig. 2:**
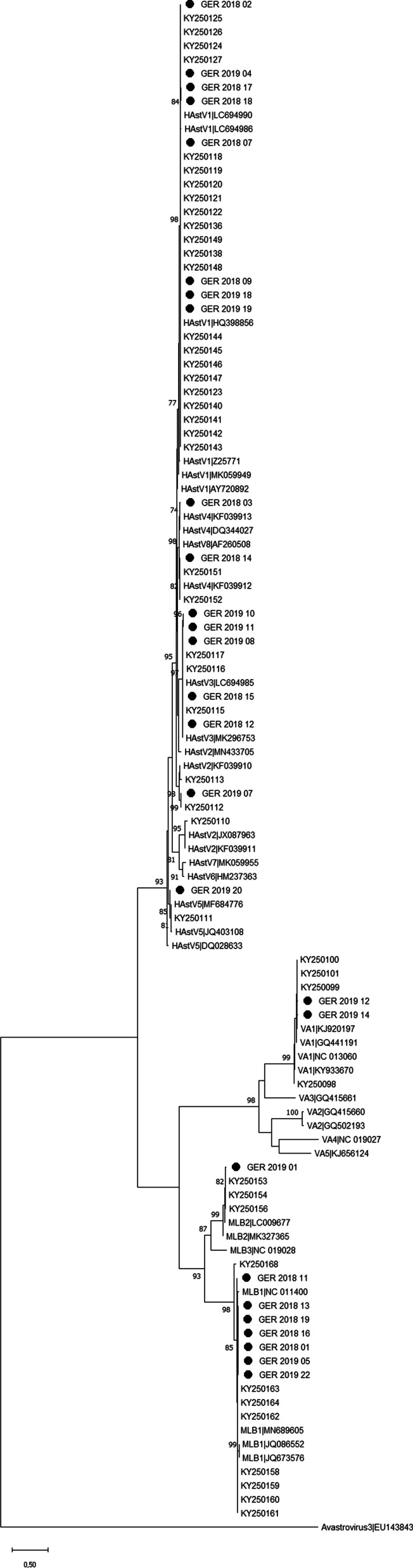
Maximum-likelihood (ML) tree of German HAstV sequences sampled 2018–2019. Lengths of sequences (ORF1 region): 307–372 bp, GenBank accession number of German HAstV sequences 2018–2019: ON886330-ON886356; the ML tree was complemented with German HAstV sequences from 2010 to 2015 (KY250098-KY250168) [7] and reference sequences. Reliability of the tree was verified by 1000 bootstrap replications

To get information about the divergence of German HAstV sequences over time, phylogenetic analysis was performed with German sequence sets from 2010 to 2015 [7] and 2018–2019 (Fig. 2). For the most prevalent genotype MLB1 (16 German sequences included) seven base differences per sequence from averaging over all sequence pairs were calculated. Base difference was also calculated in MEGA 11 within the German HAstV1 clade. An average difference of three nucleotides over all sequence pairs within 30 German HAstV1 sequences (Fig. 2) was found within a total of 335 positions in the final dataset. The evolutionary divergence among ORF1 sequences of the most predominate genotypes for the time interval 2010–2015 and 2018–2019 was low. HAstV genotypes circulating in Germany from 2010–2015 to 2018–2019 did not exhibit homogeneous clusters within each season, which is in contrast to norovirus strains circulating at the same time Germany [19]. No influence of the sampling region on clustering of German HAstV sequences was observed. It was hypothesized that the evolution of HAstV might be more affected by worldwide continuous circulation of strains than by regional influences [18]. In conclusion, the diversity of genotypes in the follow-up study was lower than in the previous time frame (2010–2015, [7]). However, circulating HAstV genotypes identified in 2018–2019 largely reflected the previous findings from 2010 to 2015.

## Material and methods

### Sample collection

In total 2645 stool samples from patients with acute gastroenteritis (sporadic cases and outbreaks) from January 2018 until December 2019 were collected and were retrospectively analyzed for human astroviruses.

### RNA extraction, HAstV detection by RT-PCR and Sanger sequencing

RNA was extracted as previously described [7]. First a pan screening PCR in the RNA-dependent RNA polymerase gene fragment was used to detect HAstV [7]. HAstV positive samples were further genotyped by a PCR adapted from Kapoor and colleagues [11, 20]. Resulting amplicons (~ 772 bp and ~ 612 bp) were sequenced by Sanger method [7].

HAstV positive samples belonging to genotypes HAstV 1–8 or MLB variants were additionally tested for recombination with PCR systems in the ORF1-ORF2 region (HAstV genotypes 1–8) or in the ORF2 region (HAstV MLB 1–3) using specific semi-nested RT-PCR systems for theses virus variants (see Additional file 1: Table S1). PCR conditions were: 30 min at 50 °C, 15 min 95 °C, followed by 30 cycles of 30 s at 94 °C, 30 s 48 °C, 30 s 68 °C for classic genotypes and 30 s 53 °C for MLB genotypes with a final cycle for 5 min at 72 °C (OneStep RT-PCR KIT (Qiagen, Germany)). For the semi-nested PCR, HotStarTaq® Master Mix Kit (Qiagen, Germany) was used under following conditions: 15 min 95 °C followed by 30 cycles of 30 s 94 °C, 30 s 52 °C, 90 s 72 °C for classic genotypes, the annealing temperature was adapted to 55 °C for MLB strains. Resulting amplicons of 854 bp for MLB and 412 bp for classic genotypes were detected by agarose gel electrophoresis and sanger sequencing were done for verification.

Sequence editing and alignments were done for phylogenetic analysis as previously described [7]. Phylogenetic analysis was performed as described in [19]. Briefly summarized, sequences were assembled and edited with Sequencher 5.4.6. Sequence alignments were performed using MAFFT algorithm with Geneious Prime 2021. In MEGA 11, best fit substitution model was calculated (Tamura 3-parameter + Gamma distribution with 5 rate categories) and modeling of a maximum-likelihood (ML) tree was done with bootstrap test of 1000 replicates [21]. The number of base differences per sequence from averaging over all sequence pairs within the genotype clade was calculated with MEGA 11 with bootstrap method as variance estimation method and 1000 replications [22]. Nucleotide sequences determined in this study were submitted to GenBank with accession numbers ON886330-ON886356.

### Statistical analyses

Fisher’s exact test was carried out with the GraphPad Prism version 9.1.0.

## Supplementary Information

**Additional file 1**.**Table S1**: ORF2-Primer sequences for genotyping HAstV classic and MLB (region ORF1-ORF2).

## Data Availability

Datasets used and/or analyzed during the current study are available from the corresponding author on reasonable request.

## Abbreviations

bp: Base pair
HAstV: Human astrovirus
ICTV: International Committee for the Taxonomy of Viruses
MAstV: Mamastrovirus
Min: Minute
ML: Maximum-likelihood
ORF: Open reading frame
RNA: Ribonucleic acid
RT-PCR: Reverse transcription polymerase chain reaction
Sec: Second
ssRNA: Single-stranded sense RNA
VPg: Viral genome-linked protein
