# Supplementary material for: Diversity of human astroviruses in Germany 2018 and 2019
Source: Virol J. 2022 Dec 21;19:221. doi: 10.1186/s12985-022-01955-3 (PMC9773458; doi:10.1186/s12985-022-01955-3)
Supplement: Supplementary file 1 — Additional file 1.Table S1: ORF2-Primer sequences for genotyping HAstV classic and MLB (region ORF1-ORF2). [file 12985_2022_1955_MOESM1_ESM.docx]

**Primer sequences for genotyping HAstV genotypes classic and MLB ( ORF1-ORF2)**

Table 1. Primer for genotyping of human astroviruses by RT-semi-nested PCR

| **HAstV genotype** | **Primer name** |  | **Sequence 5`-3** | **Localization ^a^** |
| --- | --- | --- | --- | --- |
| HAstV classic (ORF1-ORF2) | AV177 | sense | TCC TGT RCT AYC AGT TGC T | 4202-4220 |
|  | AV178 | sense | AAG CAG CTT CGT GAN TCT GG | 4288-4307 |
|  | AV180 | antisense | TRC CWG TAG CRT CCT TAA C | 4747-4709 |
| HAstV MLB (ORF2) | AV181 | sense | ATG GCT AAT GCC ART AAR GGT | 3843-3863 |
|  | AV182 | sense | TAA TMG GAA TCG CCG TCG TA | 3983-4002 |
|  | AV183 | antisense | CTG ATA AAC CAC CAT GAW GCT | 4837-1817 |

^a^ genome localization of primers are based on the sequence of NC_001943 (HAstV classic) and on the sequence of NC_019028 (HAstV MLB)
